# Clinical characteristics and patient outcomes of molecular subtypes of small cell lung cancer (SCLC)

**DOI:** 10.1186/s12957-022-02528-y

**Published:** 2022-02-27

**Authors:** Xiao-Long Ding, Yi-Ge Su, Liang Yu, Zhou-Lan Bai, Xue-Hong Bai, Xiao-Zhen Chen, Xia Yang, Ren Zhao, Jin-Xi He, Yan-Yang Wang

**Affiliations:** 1 Department of Radiation Oncology, General Hospital of Ningxia Medical University, Yinchuan, 750004 Ningxia China; 2 Graduate School, Ningxia Medical University, Yinchuan, 750004 Ningxia China; 3 Cancer Institute, Ningxia Medical University, Yinchuan, 750004 Ningxia China; 4 Department of Thoracic Surgery, General Hospital of Ningxia Medical University, Yinchuan, 750004 Ningxia China; 5 Department of Pathology, General Hospital of Ningxia Medical University, Yinchuan, 750004 Ningxia China; 6 Department of Medical Record, General Hospital of Ningxia Medical University, Yinchuan, 750004 Ningxia China

**Keywords:** Small cell lung cancer, Molecular subtype, ASCL1, NEUROD1, POU2F3, Prognosis

## Abstract

**Background:**

Recent studies have shown that according to the expression levels of achaete-scute homolog 1 (ASCL1), neurogenic differentiation factor 1 (NEUROD1), and POU class 2 homeobox 3 (POU2F3), small cell lung cancer (SCLC) can be divided into four subtypes: SCLC-A (ASCL1-dominant), SCLC-N (NEUROD1-dominant), SCLC-P (POU2F3-dominant), and SCLC-I (triple negative or SCLC-inflamed). However, there are limited data on the clinical characteristics and prognosis of molecular subtypes of SCLC.

**Methods:**

Immunohistochemistry (IHC) was used to detect the expression levels of ASCL1, NEUROD1, and POU2F3 in 53 patient samples of resectable SCLC. The subtype was defined by the differential expression of the transcription factors for ASCL1, NEUROD1, and POU2F3 or the low expression of all three factors with an inflamed gene signature (SCLC-A, SCLC-N, SCLC-P, and SCLC-I, respectively). The clinicopathological characteristics, immunological features (programmed death ligand 1 [PD-L1] expression and CD8+ tumor infiltrating lymphocyte [TIL] density), and patient outcomes of the four subtypes of SCLC were analyzed.

**Results:**

Positive ASCL1, NEUROD1, and POU2F3 staining was detected in 43 (79.2%), 27 (51.0%), and 17 (32.1%) SCLC specimens by IHC. According to the results of IHC analysis, SCLC was divided into four subtypes: SCLC-A (39.6%), SCLC-N (28.3%), SCLC-P (17.0%), and SCLC-I (15.1%). The 5-year overall survival (OS) rates of these four subtypes were 61.9%, 69.3%, 41.7%, and 85.7%, respectively (*P*=0.251). There were significant differences in smoking status among different subtypes of SCLC (*P*= 0.031). However, we did not confirm the correlation between subtypes of SCLC and other clinicopathological factors or immune profiles. Cox multivariate analysis showed that *N* stage (*P*=0.025), CD8+ TILs (*P*=0.024), Ki-67 level (*P*=0.040), and SCLC-P (*P*=0.023) were independent prognostic factors for resectable SCLC.

**Conclusions:**

Our IHC-based study validated the proposed classification of SCLC using the expression patterns of key transcriptional regulatory factors. We found that SCLC-P was associated with smokers and was one of the poor prognostic factors of limited-stage SCLC. In addition, no correlation was found between PD-L1 expression or CD8+ TIL density and SCLC subtypes.

## Introduction

Small cell lung cancer (SCLC) is a highly aggressive neuroendocrine tumor with a poor survival rate [1]. Chemotherapy and radiotherapy have been the traditional treatment modalities of SCLC for decades, but their efficacy is limited [2–4]. Immune checkpoint inhibitors (ICIs) alone or in combination show promising therapeutic efficacy in a variety of cancers, including SCLC [5, 6]. Although atezolizumab plus etoposide and carboplatin have become the new standard for the first-line treatment of extensive stage (ES)-SCLC, compared with non-small cell lung cancer (NSCLC) patients, SCLC patients benefit less from ICIs. One of the reasons for these relatively disappointing results is the heterogeneity of SCLC [7].

To improve the treatment outcomes of SCLC, Rudin et al. [8] recently proposed four molecular subtypes of SCLC based on the relative expression of the following key transcription factors: achaete-scute homolog 1 (ASCL1), neurogenic differentiation factor 1 (NEUROD1), yes-associated protein 1 (YAP1), and POU class 2 homeobox 3 (POU2F3), which were SCLC-A (ASCL1-dominant), SCLC-N (NEUROD1-dominant), SCLC-P (POU2F3-dominant), and SCLC-Y (YAP1-dominant). However, in the subsequent immunohistochemical (IHC) analysis, due to the low protein expression level of YAP1 in SCLC tissues, the SCLC-Y subtype failed to be confirmed [9]. So Gay et al. [10] redefined the subtypes of SCLC as SCLC-A, SCLC-N, SCLC-P, and SCLC-I. SCLC-I refers to the SCLC subtype with low expression levels of ASCL1, NEUROD1, and POU2F3. Although there is a substantial volume of data on SCLC subtypes in preclinical models [9, 11, 12], the clinicopathological features, immunity profiles, and treatment outcomes of these four subtypes are not clear. The clinical significance of molecular subtype classification of SCLC is worthy of further study [13].

Therefore, we studied the protein expression of target molecules related to these four subtypes in surgically resected SCLC samples. We identified four SCLC subtypes: SCLC-A, SCLC-N, SCLC-P, and SCLC-I. In addition, we comprehensively analyzed the clinicopathological features, immunity profiles, and patient outcomes of these four subtypes of SCLC. This work may contribute to the development of new treatment strategies and provide new stratification parameters for treatment selection and clinical trial design of ICIs for SCLC patients in the future.

## Materials and methods

### Patients and tissue samples

We retrospectively analyzed patients with SCLC who underwent surgery in our hospital from April 2014 to April 2020. Detailed information about the characteristics of patients was collected from electronic medical records. Most patients underwent primary tumor resection and systematic lymph node dissection. In addition to surgery, SCLC patients also received adjuvant chemotherapy with or without adjuvant thoracic radiotherapy according to the lung cancer treatment guidelines of our hospital. Prophylactic cranial irradiation (PCI) was performed after surgery, chemotherapy, and thoracic radiotherapy. Follow-up data were obtained from clinical records and telephone interviews. The surgical specimens of enrolled SCLC patients were collected for further analysis. None of the patients received radiotherapy or chemotherapy before surgery. The study protocol and the use of tissue specimens in this study were approved by the Ethics Committee of the General Hospital of Ningxia Medical University (KYLL-2020-10).

### IHC analysis

Formalin-fixed paraffin-embedded specimens were prepared, and consecutive 4-μm-thick tissue sections were cut from the specimens for IHC analysis. All sections were dewaxed in Bond Dewax Solution (Leica Microsystems, Germany) and rehydrated with graded alcohol. After dewaxing and rehydration, the sections were heated under high pressure at 100°C for 20 min for antigen repair. The sections were then incubated with 3% hydrogen peroxide solution for 10 min to block the activity of endogenous peroxidase. After that, all sections were incubated with the primary antibody overnight at 4°C, washed with phosphate-buffered saline, and incubated with horseradish peroxidase-conjugated goat anti-mouse/rabbit IgG detection antibody. Finally, all sections were visualized with 3,3’-diaminobenzidine (DAB) after counterstaining with hematoxylin. Leica Application Suite (Leica Microsystems, Germany) was used for image acquisition. The following antibodies were used for IHC staining: ASCL1 (Abcam, UK), NEUROD1 (Abcam, UK), POU2F3 (Abcam, UK), programmed death ligand 1 (PD-L1) (Abcam, UK), and CD8 (ZSGB-Bio, China).

Two pathologists examined the stained sections. The subtypes of SCLC were determined by the H-score of ASCL1, NEUROD1, and POU2F3. The H-score was developed using the method described previously based on the staining intensity and the percentage of positive cells of the indicated protein [14]. Tumor cell membrane staining was considered to be PD-L1 positive. A semiquantitative scoring method was used to evaluate the expression level of PD-L1: 1 = <1% of cells were stained, 2 = 1–5%, 3 = 6–10%, 4 = 11–25%, 5 = 26–50%, and 6 = >50%. Tumors with a score of ≥2 were defined as having high expression of PD-L1 [15]. The cutoff value for high/low CD8+ tumor infiltrating lymphocyte (TIL) density was 30% [16]. To determine the dominant phenotype of patients with ASCL1, NEUROD1, or POU2F3 expression, cases in which ASCL1 was higher than NEUROD1 or POU2F3 were considered to be ASCL1-dominant and vice versa. SCLC-I was defined as an SCLC subtype with negative expression of ASCL1, NEUROD1, and POU2F3 [9, 10].

### Statistical analysis

The chi-square test or Fisher’s exact test was used to analyze the correlation between subtypes of SCLC and clinicopathological variables and immunity profiles. Overall survival (OS) was defined as the time from diagnosis of SCLC to death or censoring. The survival time of patients was estimated by the Kaplan–Meier method. The survival rate of each group was compared by log-rank tests. Cox’s proportional hazard model was used for univariate and multivariate survival analysis. When the *p* value was less than 0.05, the difference was considered to be statistically significant. All statistical analyses were performed based on SPSS v.20.0 (SPSS Inc., Chicago, USA) for Windows and GraphPad Prism v.7.0 (GraphPad Software, Inc., La Jolla, USA).

## Results

### Patient characteristics

Fifty-three patients were finally analyzed, and 36 (67.9%) patients were male. The median age of all patients was 59 years (range, 35–73 years), and 29 (54.7%) patients were never-smokers. According to the 8th edition of the International Association for the Study of Lung Cancer TNM Staging System, 40 (75.4%) patients were classified as stage I–II disease, and 13 (24.6%) patients were classified as stage III disease. Thirty-nine (73.6%) patients received adjuvant chemotherapy, 15 (28.3%) patients received postoperative radiotherapy, and 14 (26.4%) patients received PCI treatment. The detailed patient characteristics are described in Table 1.

**Table 1:** Patient’s characteristics

Characteristic	SCLC-A	SCLC-N	SCLC-P	SCLC-I	*P* value
Gender					
Male	14	11	7	4	
Female	7	4	2	4	0.650
Age					
≥65	7	5	2	2	
<65	14	10	7	6	0.949
Smoking status					
Current/former smoker	8	6	8	2	
Non-smoker	13	9	1	6	0.031
Tumor location					
Upper or middle lobe	9	8	4	4	
Lower lobe	12	7	5	4	0.978
Histology					
Combined	1	1	3	1	
Pure	20	14	6	7	0.142
TNM stage					
I-II	16	12	5	7	
III	5	3	4	1	0.495
T stage					
T1-2	18	14	6	6	
T3	3	1	3	2	0.296
N stage					
N0-1	17	12	6	7	
N2	4	3	3	1	0.786
Ki-67 status					
High	5	6	3	3	
Low	16	9	6	5	0.743
PD-L1 status					
High	7	2	1	1	
Low	14	13	8	7	0.455
CD8 status					
High	9	6	4	2	
Low	12	9	5	6	0.864

*Abbreviations*: *SCLC* small cell lung cancer, *SCLC-A* ASCL1-dominant SCLC, *SCLC-N* NEUROD1-dominant SCLC, *SCLC-P* POU2F3-dominant SCLC, *SCLC-I* triple negative or SCLC-inflamed SCLC, *TNM* tumor-node-metastasis, *PD-L1* programmed death-ligand 1

### Define subtypes of SCLC

**Fig. 1:**
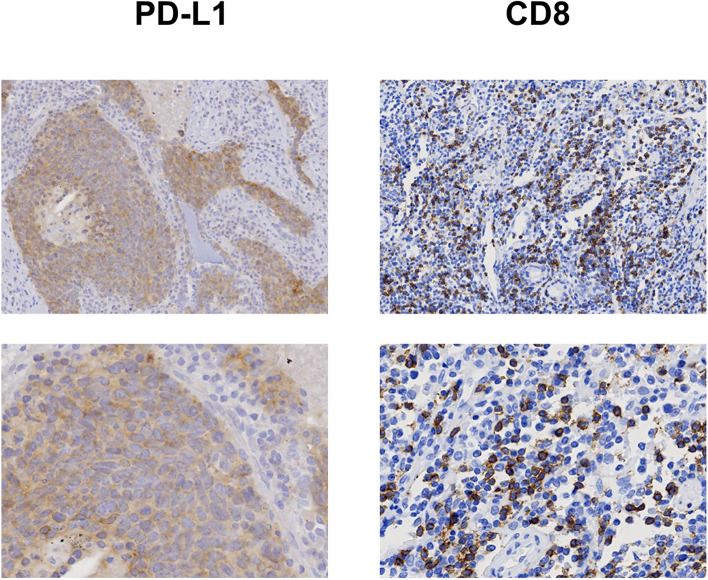
Representative images of PD-L1 expression and CD8+ tumor infiltrating lymphocytes (TILs) in patients with small cell lung cancer (SCLC), as determined by immunohistochemistry (IHC). The first line 100× magnification. The second line 400× magnification

A total of 53 samples from the primary lung tumors were stained and analyzed by IHC. Both ASCL1 and NEUROD1 were expressed in the nuclei of tumor cells. POU2F3 was predominantly expressed in the cytoplasm of tumor cells. Positive ASCL1, NEUROD1, and POU2F3 staining were detected in 43 (79.2%), 27 (51.0%), and 17 (32.1%) SCLC specimens, respectively. The mean H-scores of ASCL1-, NEUROD1-, and POU2F3-positive cases were 109.7 (range: 30.0–240.0), 50.9 (range: 2.0–210.0), and 157 (range: 0–300.0), respectively. PD-L1 immunostaining was mainly localized in the plasma membrane of SCLC cells. The percentages of patients with PD-L1 expression scores of 1, 2, 3, 4, 5, and 6 were 22.2%, 66.7%, 0%, 0%, 11.1%, and 0, respectively. Higher expression of PD-L1 in tumor cells was found in 11 (20.8%) cases. Higher CD8+ TIL densities were observed in 21 (39.6%) SCLC specimens. Representative stained images of PD-L1 and CD8 are shown in Fig. 1.

**Fig. 2:**
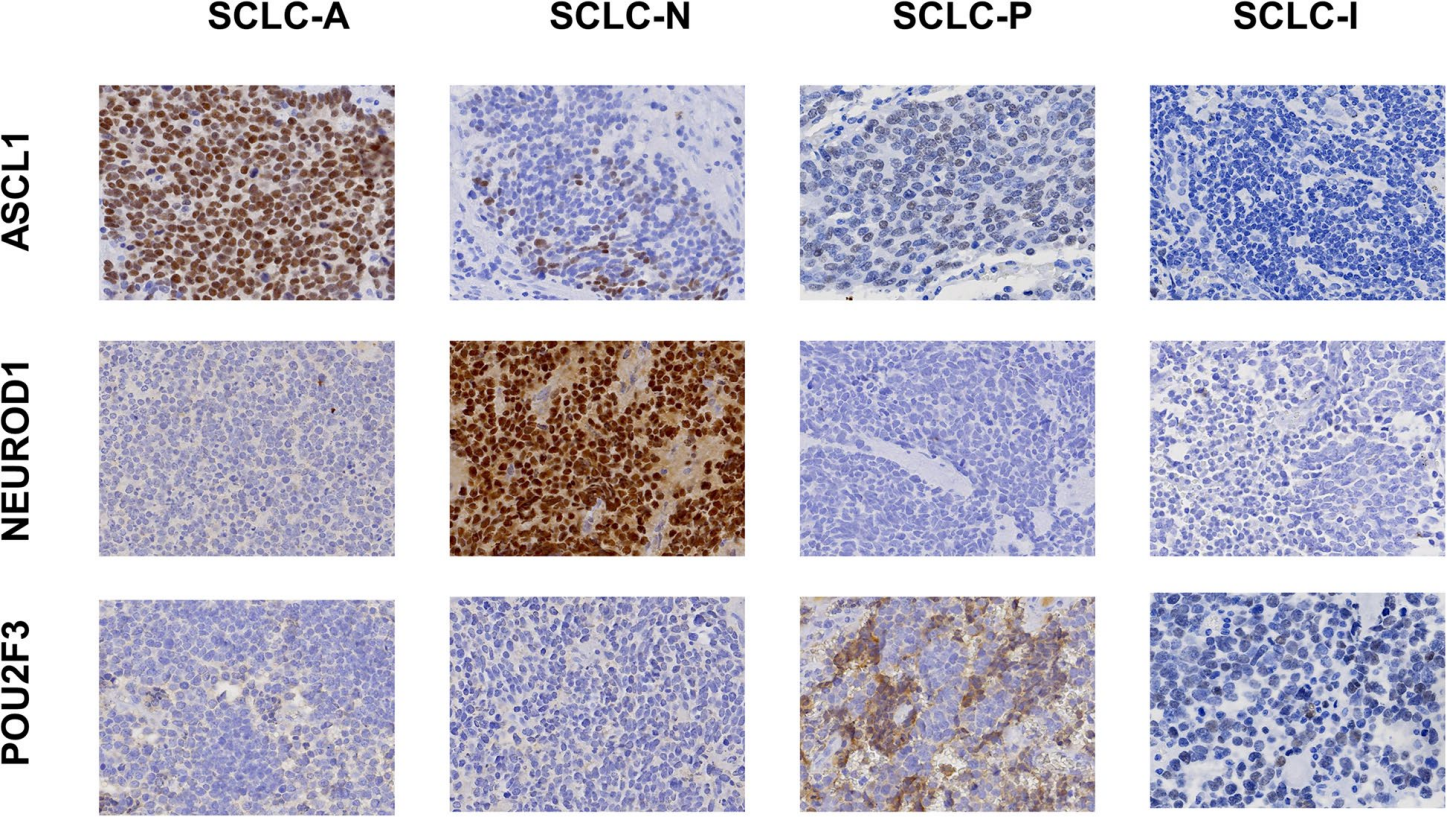
Representative immunohistochemistry (IHC) images of small cell lung cancer (SCLC) subtypes as defined by ASCL1, NEUROD1, and POU2F3 expression

**Fig. 3:**
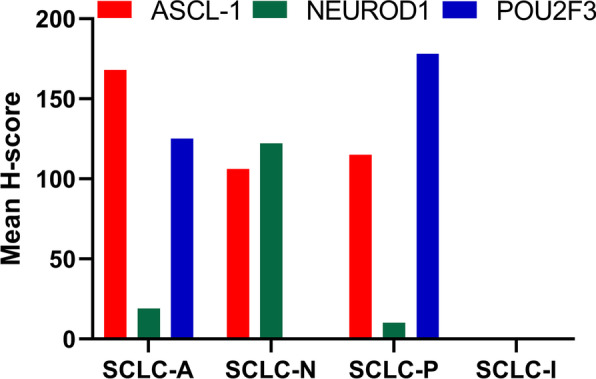
The mean H-score of ASCL1, NEUROD1, and POU2F3 in small cell lung cancer (SCLC) subtypes

On the basis of the results of ASCL1, NEUROD1, and POU2F3 IHC analyses (Fig. 2), we defined four subtypes of SCLC: SCLC-A, SCLC-N, SCLC-P, and SCLC-I. The distribution of subtypes was as follows: SCLC-A 39.6%, SCLC-N 28.3%, SCLC-P 17.0%, and SCLC-I 15.1%. The mean H-scores of ASCL1, NEUROD1, and POU2F3 in different subtypes are shown in Fig. 3.

### Clinical characteristics, immune profiles, and survival outcomes of different SCLC subtypes

Next, we analyzed the clinical characteristics, immune profiles, and survival outcomes of these four SCLC subtypes. The results showed that there were significant differences in smoking status among different subtypes of SCLC (*P*=0.031). The proportion of current/former smokers was the highest in the SCLC-P subtype. However, SCLC subtypes were not associated with other clinical parameters, such as sex, age, tumor location, histology, TNM stage, tumor size, lymph node metastasis, or Ki-67 level. In addition, the correlation between SCLC subtypes and immune profiles (such as PD-L1 expression level or CD8+ TIL density) was not confirmed in this study.

**Fig. 4:**
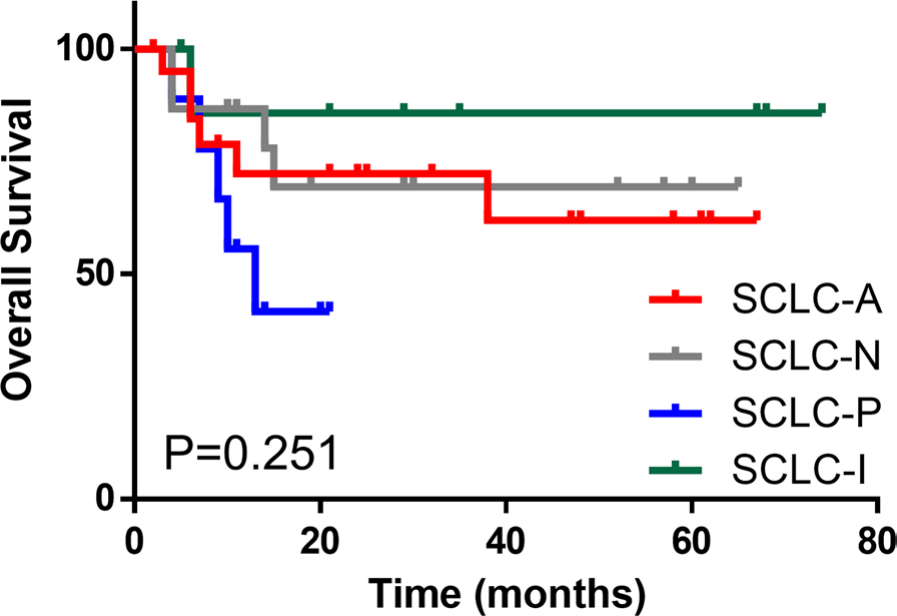
Kaplan-Meier analysis of overall survival (OS) in patients with different subtypes of small cell lung cancer (SCLC)

Subsequently, we examined the survival outcomes of these distinct subtypes. The follow-up period was 2.0–74.0 months, with a median of 29.0 months. There was no significant difference in OS among the four subtypes (*P*=0.251) (Fig. 4). However, a consistent trend was noted toward a better outcome for the SCLC-I subtype. The 5-year OS rates of the SCLC-A, SCLC-N, SCLC-P, and SCLC-I subtypes were 61.9%, 69.3%, 41.7%, and 85.7%, respectively.

### Prognostic factors of surgically resectable SCLC patients

**Fig. 5:**
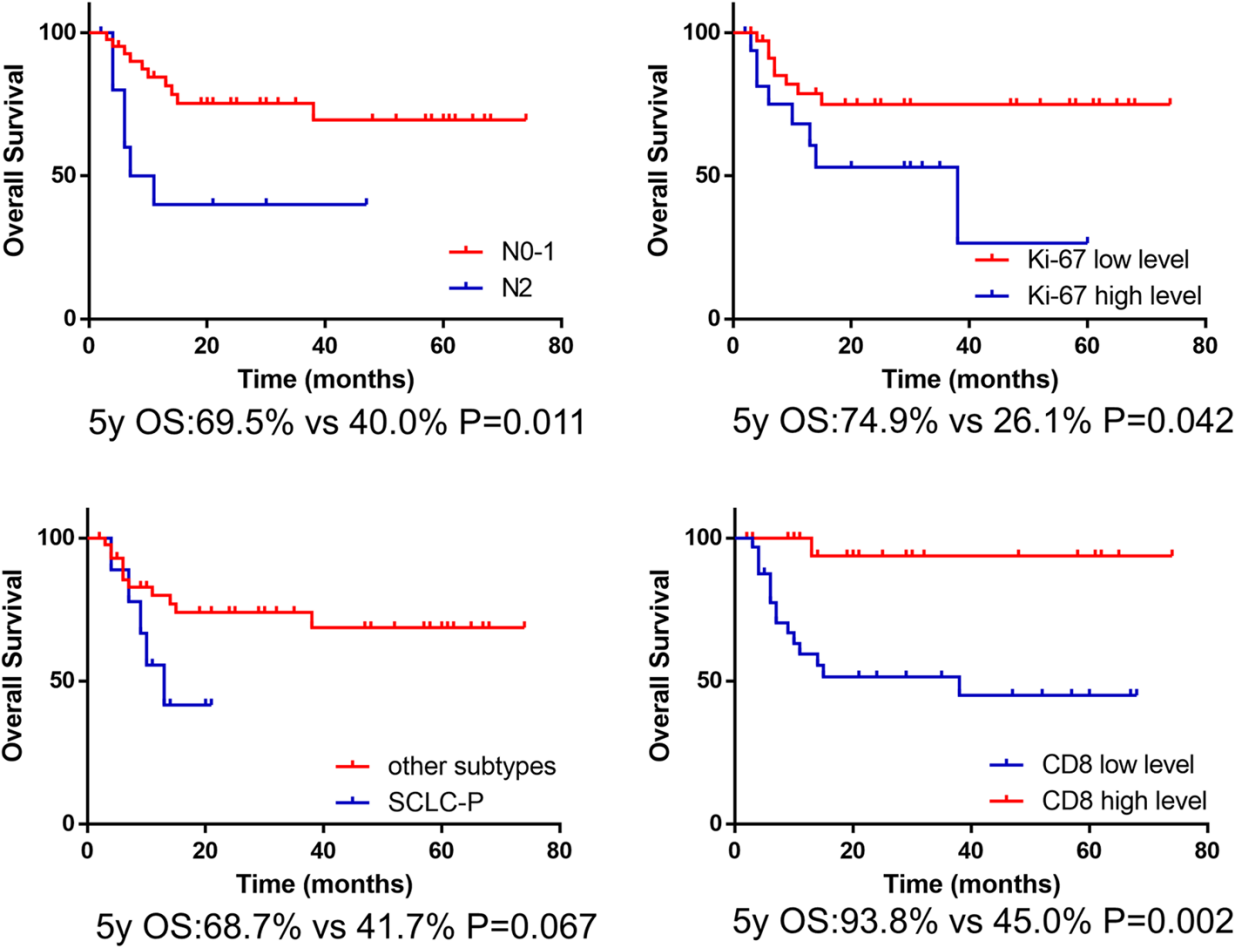
Kaplan-Meier analysis of overall survival (OS) based on lymph node metastasis, Ki-67, SCLC-P subtype, or CD8+ tumor infiltrating lymphocytes (TILs) density expression in patients with small cell lung cancer (SCLC)

The prognostic factors of surgically resectable SCLC were analyzed by Cox analysis. Univariate analysis showed that TNM stage (*P*=0.007), *T* stage (*P*=0.025), *N* stage (*P*=0.017), CD8+ TILs (*P*=0.015), adjuvant chemotherapy (*P*=0.036), and adjuvant radiotherapy (*P*=0.042) were associated with prolonged OS. The Ki-67 level and SCLC-P subtype reached marginal statistical significance in the univariate analysis (Table 2). Multivariate analysis showed that *N* stage (*P*=0.025), CD8+ TILs (*P*=0.024), Ki-67 level (*P*=0.040), and SCLC-P (*P*=0.023) were independent prognostic factors of resectable SCLC patients (Table 2). The Kaplan–Meier curves for OS according to these four prognostic factors, which were demonstrated in multivariate analysis, are shown in Fig. 5.

**Table 2:** Univariate and multivariate survival analysis for overall survival

Variables	Univariate analysis			Multivariate analysis		
	HR	95% CI	*P* value	HR	95% CI	*P* value
Gender	1.523	0.489–4.746	0.468			
Age	0.284	0.064–1.250	0.096			
Smoking status	1.439	0.522–3.970	0.482			
Tumor location	1.313	0.492–3.499	0.587			
Histology	1.876	0.533–6.595	0.327			
TNM stage	4.021	1.471–10.994	0.007	0.035	0.001–2.419	0.121
T stage	3.373	1.162–9.795	0.025	3.428	0.430–27.346	0.245
N stage	3.467	1.244–9.664	0.017	31.714	1.543–652.031	0.025
Ki-67 status	2.650	0.989–7.101	0.053	3.890	1.064–14.228	0.040
SCLC-A	0.985	0.357–2.718	0.977			
SCLC-N	0.777	0.250–2.412	0.662			
SCLC-P	2.617	0.887–7.727	0.082	7.582	1.321–43.504	0.023
SCLC-I	0.326	0.043–2.470	0.278			
PD-L1 status	0.226	0.030–1.716	0.151			
CD8 status	0.081	0.011–0.617	0.015	0.070	0.007–0.706	0.024
Adjuvant chemotherapy	0.351	0.131–0.936	0.036	0.369	0.090–1.514	0.166
Adjuvant radiotherapy	0.122	0.016–0.924	0.042	0.102	0.008–1.322	0.081

*Abbreviations*: *HR* hazard ratio, *CI* confidence interval, *TNM* tumor-node-metastasis, *SCLC* small cell lung cancer, *SCLC-A* ASCL1-dominant SCLC, *SCLC-N* NEUROD1-dominant SCLC, *SCLC-P* POU2F3-dominant SCLC, *SCLC-I* triple negative or SCLC-inflamed SCLC, *PD-L1* programmed death-ligand 1

## Discussion

Due to the understanding of SCLC biology in recent years, it was suggested that SCLC could be divided into different subtypes according to the expression levels of ASCL1, NEUROD1, and POU2F3 [8]. In SCLC, ASCL1 activates neuroendocrine differentiation and regulates stemness, cell cycle progression, and mitosis, thus maintaining tumor development and survival [17, 18]. NEUROD1 is also involved in the neuroendocrine differentiation of SCLC. It targets MYC and promotes the development of slow-growing neurogenic differentiated tumors [17, 19]. POU2F3 is a master regulator that is essential for the generation, chemosensory, and immune functions of specialized tuft cells in the respiratory tract [20–22]. These three molecules play a crucial role in driving the malignant biological behavior of SCLC. In this study, we found that ASCL1, NEUROD1, and POU2F3 were expressed in 79.2%, 50.9%, and 32.1% of tumors at any level, respectively. This information is consistent with the results of a recent report [9]. On the basis of these three markers, the proportion of SCLC subtypes in our cohort was 39.6% SCLC-A, 28.3% SCLC-N, 17.0% SCLC-P, and 15.1% SCLC-I, which was similar to the distribution previously reported [10, 23].

Biologically, SCLC-A tumors have typical morphological features, showing high expression of neuroendocrine markers [24]. Compared with SCLC-A, the SCLC-N subtype has a different gene expression profile, while neuroendocrine gene and delta-like ligand 3 (DLL3) expression are downregulated [25, 26]. These two subtypes seem to be immunologically cold due to lower human leukocyte antigen (HLA) gene expression and T cell infiltration [27]. The SCLC-P subtype may originate from cells different from other SCLC subtypes. The gene expression profile (GEP) score of T cell inflammation of the SCLC-P subtype is moderately high, but the expression of cancer testis antigen (CTA) is the highest, indicating that this subtype of tumor is poorly differentiated and is unlikely to respond to ICIs alone [27]. SCLC-I shows epithelial–mesenchymal transition (EMT) and inflammatory phenotypes, which are related to the activation of interferon-γ and the expression of genes related to immune checkpoints [28]. It is suggested that the SCLC-I subtype may be a key subtype of SCLC patients who benefit from ICIs. To further illustrate the features of SCLC subtypes, we studied the clinicopathological parameters and survival rates of these subtypes. The results showed that only smoking status was related to the SCLC subtype. The number of smokers with the SCLC-P subtype was greater than that of smokers of the other subtypes. However, it is worth mentioning that, due to the low smoking rate of female patients and Hui patients, the proportion of nonsmoking patients included in this study was higher. In addition, due to the higher proportion of TNM stage I and II in the cohort

The 5-year survival rates of SCLC-A, SCLC-N, SCLC-P, and SCLC-I were 61.9%, 69.3%, 41.7%, and 85.7%, respectively. Moreover, a long follow-up period is required to fully assess the 5-year survival rate of different subtypes.

Increasing evidence shows that blocking immune checkpoints, such as programmed death 1 (PD-1)/PD-L1 or cytotoxic T lymphocyte-associated antigen 4 (CTLA-4), has a certain antitumor effect on patients with ES-SCLC [5, 6, 29]. However, only a small number of patients with SCLC have shown durable benefits from ICIs. PD-L1 expression and CD8+ TIL density, as the most important antitumor immune response factors, often determine the efficacy of anti-PD-1/PD-L1 in various solid tumors [30, 31]. To better select the target population that may benefit from ICIs, we analyzed the correlation between different SCLC subtypes and PD-L1 expression or CD8+ TIL density in subsequent studies. The positive expression rate of PD-L1 was 20.8% in this analysis, which was lower than that reported in NSCLC. We also demonstrated that the proportion of high TIL infiltration of CD8 was 39.6%. However, we did not observe any association between PD-L1 expression or CD8+ TIL density and SCLC subtypes in the current cohort, mainly due to the small sample size.

In the last part of this study, we used Cox analysis to screen the prognostic factors of patients with surgically resectable SCLC. In multivariate analysis, *N* stage, CD8+ TILs, Ki-67 level, and SCLC-P were selected as independent prognostic factors for surgically resectable SCLC. It is suggested that SCLC classification could at least screen subtypes with poor prognosis, such as SCLC-P.

This study has several limitations. First, the retrospective design of this study increased the risk of patient selection bias. Second, the number of patients enrolled in the study was relatively small. Third, due to the high proportion of patients with TNM stages I and II in the cohort (75.4%), the overall survival rate of each subtype is satisfactory. This should be noted when comparing with other studies. Four, the study population included only limited-stage disease with primary lung cancer specimens, suggesting that these findings should be interpreted with caution. Finally, mature survival information was limited because the follow-up time in our study was not long enough to fully assess 5-year survival rates. Future large-sample prospective studies are warranted to overcome these limitations and validate our results.

In summary, our IHC-based study validated the proposed classification of SCLC using the expression patterns of key transcriptional regulatory factors. We found that SCLC-P was associated with smokers and was one of the poor prognostic factors of limited-stage SCLC. In addition, no correlation was found between PD-L1 expression or CD8+ TIL density and SCLC subtypes.

## Data Availability

The datasets used and/or analyzed during the current study are available from the corresponding authors on reasonable request.

## Abbreviations

ASCL1: Achaete-scute homolog 1
CTA: Cancer testis antigen
CTLA-4: Cytotoxic T-lymphocyte-associated antigen 4
EMT: Epithelial–mesenchymal transition
ES: Extensive stage
GEP: Gene expression profile
HLA: Human leukocyte antigen
ICIs: Immune checkpoint inhibitors
IHC: Immunohistochemical
NEUROD1: Neurogenic differentiation factor 1
NSCLC: Non-small cell lung cancer
OS: Overall survival
PCI: Prophylactic cranial irradiation
PD-L1: Programmed death ligand 1
POU2F3: POU class 2 homeobox 3
SCLC: Small cell lung cancer
TILs: Tumor infiltrating lymphocytes
YAP1: Yes-associated protein 1

## References

[1] Rudin CM, Brambilla E, Faivre-Finn C, Sage J (2021). Small-cell lung cancer. Nat Rev Dis Primers.

[2] Dingemans AC, Fruh M, Ardizzoni A, Besse B, Faivre-Finn C, Hendriks LE (2021). Small-cell lung cancer: ESMO Clinical Practice Guidelines for diagnosis, treatment and follow-up(). Ann Oncol.

[3] Faivre-Finn C, Snee M, Ashcroft L, Appel W, Barlesi F, Bhatnagar A (2017). Concurrent once-daily versus twice-daily chemoradiotherapy in patients with limited-stage small-cell lung cancer (CONVERT): an open-label, phase 3, randomised, superiority trial. Lancet Oncol.

[4] Slotman BJ, van Tinteren H, Praag JO, Knegjens JL, El Sharouni SY, Hatton M (2015). Use of thoracic radiotherapy for extensive stage small-cell lung cancer: a phase 3 randomised controlled trial. Lancet.

[5] Horn L, Mansfield AS, Szczesna A, Havel L, Krzakowski M, Hochmair MJ (2018). First-line Atezolizumab plus chemotherapy in extensive-stage small-cell lung cancer. N Engl J Med.

[6] Paz-Ares L, Dvorkin M, Chen Y, Reinmuth N, Hotta K, Trukhin D (2019). Durvalumab plus platinum-etoposide versus platinum-etoposide in first-line treatment of extensive-stage small-cell lung cancer (CASPIAN): a randomised, controlled, open-label, phase 3 trial. Lancet.

[7] Shue YT, Lim JS, Sage J (2018). Tumor heterogeneity in small cell lung cancer defined and investigated in pre-clinical mouse models. Transl Lung Cancer Res.

[8] Rudin CM, Poirier JT, Byers LA, Dive C, Dowlati A, George J (2019). Molecular subtypes of small cell lung cancer: a synthesis of human and mouse model data. Nat Rev Cancer.

[9] Baine MK, Hsieh MS, Lai WV, Egger JV, Jungbluth AA, Daneshbod Y (2020). SCLC Subtypes Defined by ASCL1, NEUROD1, POU2F3, and YAP1: A comprehensive immunohistochemical and histopathologic characterization. J Thorac Oncol.

[10] Gay CM, Stewart CA, Park EM, Diao L, Groves SM, Heeke S (2021). Patterns of transcription factor programs and immune pathway activation define four major subtypes of SCLC with distinct therapeutic vulnerabilities. Cancer Cell.

[11] Ireland AS, Micinski AM, Kastner DW, Guo B, Wait SJ, Spainhower KB (2020). MYC drives temporal evolution of small cell lung cancer subtypes by reprogramming neuroendocrine fate. Cancer Cell.

[12] Mukhopadhyay S, Dermawan JK, Lanigan CP, Farver CF (2019). Insulinoma-associated protein 1 (INSM1) is a sensitive and highly specific marker of neuroendocrine differentiation in primary lung neoplasms: an immunohistochemical study of 345 cases, including 292 whole-tissue sections. Mod Pathol.

[13] Schwendenwein A, Megyesfalvi Z, Barany N, Valko Z, Bugyik E, Lang C (2021). Molecular profiles of small cell lung cancer subtypes: therapeutic implications. Mol Ther Oncolytics.

[14] Fedchenko N, Reifenrath J (2014). Different approaches for interpretation and reporting of immunohistochemistry analysis results in the bone tissue - a review. Diagn Pathol.

[15] Kasahara N, Kaira K, Yamaguchi K, Masubuchi H, Tsurumaki H, Hara K (2019). Fluorodeoxyglucose uptake is associated with low tumor-infiltrating lymphocyte levels in patients with small cell lung cancer. Lung Cancer.

[16] Sun C, Zhang L, Zhang W, Liu Y, Chen B, Zhao S (2020). Expression of PD-1 and PD-L1 on tumor-infiltrating lymphocytes predicts prognosis in patients with small-cell lung cancer. Onco Targets Ther.

[17] Borromeo MD, Savage TK, Kollipara RK, He M, Augustyn A, Osborne JK (2016). ASCL1 and NEUROD1 reveal heterogeneity in pulmonary neuroendocrine tumors and regulate distinct genetic programs. Cell Rep.

[18] Lantuejoul S, Fernandez-Cuesta L, Damiola F, Girard N, McLeer A (2020). New molecular classification of large cell neuroendocrine carcinoma and small cell lung carcinoma with potential therapeutic impacts. Transl Lung Cancer Res.

[19] Mollaoglu G, Guthrie MR, Bohm S, Bragelmann J, Can I, Ballieu PM (2017). MYC drives progression of small cell lung cancer to a variant neuroendocrine subtype with vulnerability to aurora kinase inhibition. Cancer Cell.

[20] O'Leary CE, Schneider C, Locksley RM (2019). Tuft cells-systemically dispersed sensory epithelia integrating immune and neural circuitry. Annu Rev Immunol.

[21] von Moltke J, Ji M, Liang HE, Locksley RM (2016). Tuft-cell-derived IL-25 regulates an intestinal ILC2-epithelial response circuit. Nature.

[22] Yamashita J, Ohmoto M, Yamaguchi T, Matsumoto I, Hirota J (2017). Skn-1a/Pou2f3 functions as a master regulator to generate Trpm5-expressing chemosensory cells in mice. PLoS One.

[23] George J, Lim JS, Jang SJ, Cun Y, Ozretic L, Kong G (2015). Comprehensive genomic profiles of small cell lung cancer. Nature.

[24] Drapkin BJ, Rudin CM (2021). Advances in small-cell lung cancer (SCLC) translational research. Cold Spring Harb Perspect Med.

[25] Cardnell RJ, Li L, Sen T, Bara R, Tong P, Fujimoto J (2017). Protein expression of TTF1 and cMYC define distinct molecular subgroups of small cell lung cancer with unique vulnerabilities to aurora kinase inhibition, DLL3 targeting, and other targeted therapies. Oncotarget.

[26] Saunders LR, Bankovich AJ, Anderson WC, Aujay MA, Bheddah S, Black K (2015). A DLL3-targeted antibody-drug conjugate eradicates high-grade pulmonary neuroendocrine tumor-initiating cells in vivo. Sci Transl Med.

[27] Owonikoko TK, Dwivedi B, Chen Z, Zhang C, Barwick B, Ernani V (2021). YAP1 Expression in SCLC defines a distinct subtype with T-cell-inflamed phenotype. J Thorac Oncol.

[28] Mak MP, Tong P, Diao L, Cardnell RJ, Gibbons DL, William WN (2016). A patient-derived, pan-cancer emt signature identifies global molecular alterations and immune target enrichment following epithelial-to-mesenchymal transition. Clin Cancer Res.

[29] Arriola E, Wheater M, Galea I, Cross N, Maishman T, Hamid D (2016). Outcome and biomarker analysis from a multicenter phase 2 study of ipilimumab in combination with carboplatin and etoposide as first-line therapy for extensive-stage SCLC. J Thorac Oncol.

[30] Kim TK, Herbst RS, Chen L (2018). Defining and understanding adaptive resistance in cancer immunotherapy. Trends Immunol.

[31] Apetoh L, Smyth MJ, Drake CG, Abastado JP, Apte RN, Ayyoub M (2015). Consensus nomenclature for CD8(+) T cell phenotypes in cancer. Oncoimmunology.

